# Supplementary material for: AMAISE: a machine learning approach to index-free sequence enrichment
Source: Commun Biol. 2022 Jun 9;5:568. doi: 10.1038/s42003-022-03498-3 (PMC9184628; doi:10.1038/s42003-022-03498-3)
Supplement: Supplementary file 2 — Supplementary Information [file 42003_2022_3498_MOESM2_ESM.pdf]

Supplementary Notes

Supplementary Note 1: Comparison to Methods with Pan-Genome Indices

When using a pan genome index to classify a read set with 99% host data, 1% bacterial and fungal data, Minimap2's accuracy did not improve. It decreased from 84% to 33%. While k-mer based indices can include genetic variation via pan genomes, they cannot effectively include variation via large amounts of sequenced data from various sequencing technologies. A benefit of AMAISE compared to k-mer based approaches it that it can learn not just from reference genomes but from sequenced data that has not been aligned. Alignment inherently removes variation that can be caused by sequencing errors, making any method that learns from reference genomes less robust to sequencing errors. Thus, AMAISE's advantage is not that it has a "peek" at general genetic variation, but that it can learn from sequenced reads that have not been aligned.

Supplementary Note 2: Comparison on Non-Human Hosts

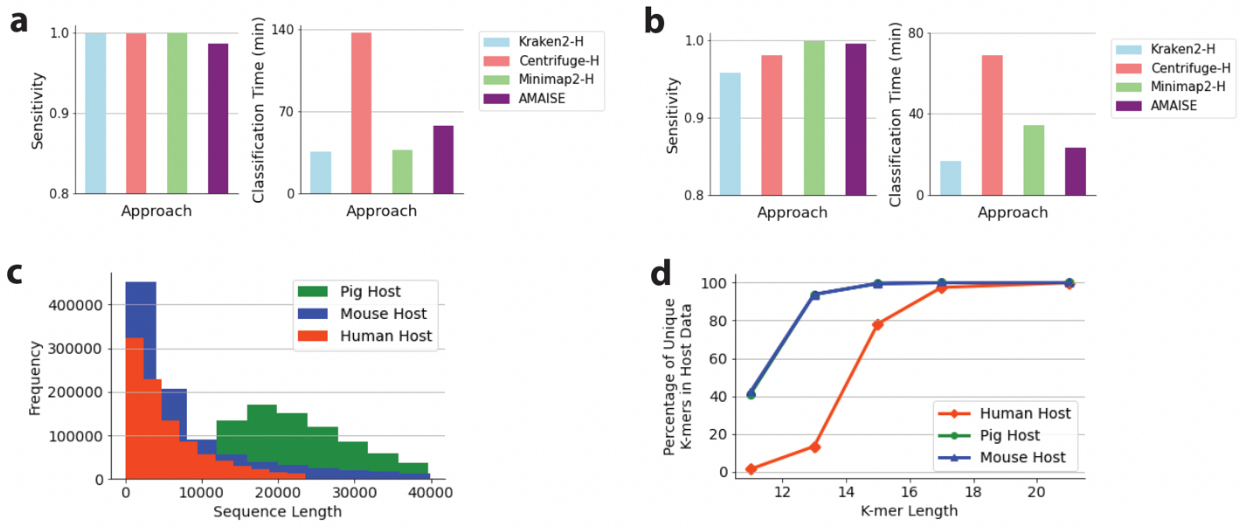

Supplementary Figure S1. a) Sensitivity and classification time on test set with pig host. On data with a pig host, AMAISE achieved comparable sensitivity with much lower classification time than Centrifuge. b) Sensitivity and classification time on test set with mouse host. AMAISE was faster than Minimap2 and Centrifuge at classifying the data with a mouse host and had a higher sensitivity than Kraken2 and Centrifuge on these data. c) Length of test set with human host, pig host, and mouse host. The samples with pig and mouse hosts, on average, contained longer reads than the samples with a human host. d) Percentage of unique host k-mers in test set with

human host, pig host, and mouse host. The samples with pig and mouse hosts contain more unique k-mers in the host data compared to the samples with a human host.

Supplementary Note 3: Comparison on Microbes Similar to Host

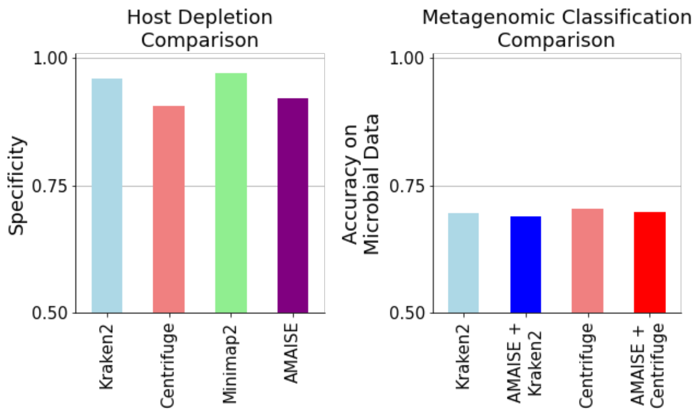

Supplementary Figure S2. The two metrics that changed when adding viruses to our test set were specificity and microbial accuracies. AMAISE achieved comparable specificity to Centrifuge, and lower specificity than Kraken2 and Minimap2 (left figure). However, when used in a pipeline with Kraken2, AMAISE achieved the same microbial accuracy as Kraken2 (right figure).

Supplementary Note 4: Metagenomic Classification Methods with No Host Reference

We hypothesized that metagenomic classification tools would not be effective at classifying host and microbial sequences when their reference databases do not have host DNA in them, meaning that AMAISE would need to be used before a metagenomic classification tool without host DNA in its reference database. To test this hypothesis, we assessed the ability of Kraken2-M, Centrifuge-M, Kraken2-HM, and Centrifuge-HM to accurately classify 10,000 randomly selected Nanopore sequences with the ground truth label of 'Homo sapiens.' We consider an accurate classification when host DNA was in the reference database to be the classification label 'Homo sapiens.' We consider an accurate classification when host DNA was not in the reference database to be the classification label 'Unclassified' or a higher taxonomic label that could be used to identify humans. The results of our experiment are in supplementary Figure

S3.

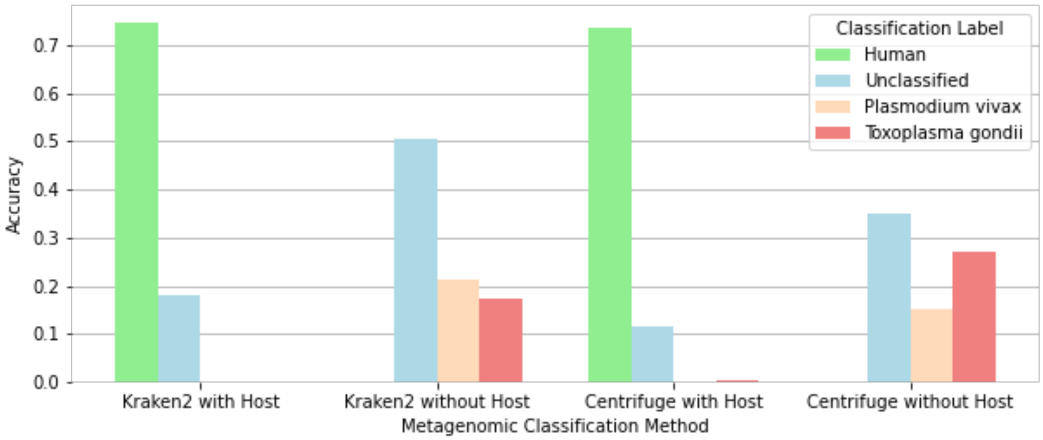

Supplementary Figure S3. A histogram showing the accuracy of Kraken2-M, Centrifuge-M, Kraken2-HM, and Centrifuge-HM on 10,000 Nanopore sequences with the ground truth label of 'Homo sapiens.'

When we input 10,000 Nanopore sequences with the ground truth label of 'Homo sapiens' into Kraken2-HM, 93% of those sequences were classified as 'Homo sapiens' or left unclassified. When we input those same sequences into Kraken2-M, only 50% of those sequences were left unclassified. The rest were classified as microbial organisms. 21% of sequences were classified as being from the protozoa Plasmodium vivax, and 17% of sequences were classified as being from the protozoa Toxoplasma gondii. Thus, Kraken2-M was unable to either leave most human sequences as unclassified or classified at a higher taxonomic level than species.

We performed the same experiment with Centrifuge. Centrifuge-HM was similarly able to classify 85% of the human sequences as 'Homo sapiens' or leave them unclassified. However, Centrifuge-M leaves only 35% of the sequences unclassified, and incorrectly identifies 27% of the sequence as being from Toxoplasma gondii and 15% of the sequences as being from Plasmodium vivax.

Thus, for Kraken2-M and Centrifuge-M to accurately classify and discard host sequences, they must be used in a pipeline with a host depletion method.

Supplementary Note 5: Full Metagenomic Classification Pipeline Comparison

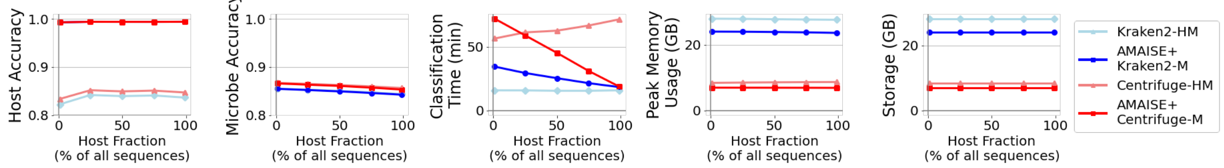

Supplementary *Figure S4: Performance of Centrifuge-HM, Kraken2-HM, AMAISE + Centrifuge-M, and AMAISE + Kraken2-M across samples that varied in terms of host percentage. Across samples that varied in terms of host percentage, the pipelines that included AMAISE consistently achieved higher host accuracies and microbial accuracies while requiring less peak memory usage and storage and remaining competitive with respect to classification time.*

Supplementary Note 6: Methods Tables

Supplementary Table 1. Hyperparameters used to determine the optimal architecture for AMAISE

Parameter	Values
Number of Filters	{16, 32, 64, 128}
Size of Filter	{3, 5, 10, 15, 21}
Number of Convolutional Layers	{1, 2, 3, 4, 5}
Number of Fully Connected Layers	{1, 2, 3}
Size of Average Pooling Between Convolutional Layers	{1, 2, 3, 4, 5}
Dropout Between Convolutional Layers	{0, 0.1, 0.2, 0.3, 0.4}
Learning Rate	{1e-2, 1e-3, 1e-4, 1e-5}
Weight Decay for L2 Regularization	{1e-5, 1e-6, 1e-7}

Supplementary Table 2. The thresholds that AMAISE uses to threshold probabilities to classification labels. If the output probability is greater than the threshold, then the corresponding input is considered to be from a host. Else, the corresponding input is considered to be from a microbe.

Sequence Length	AMAISE's Thresholds
25	0.31
50	0.41
100	0.54
150	0.62
200	0.70
250	0.64
300	0.66
500	0.64
1000	0.47
5000	0.48
10000	0.46
