# Supplementary material for: AMAISE: a machine learning approach to index-free sequence enrichment
Source: Commun Biol. 2022 Jun 9;5:568. doi: 10.1038/s42003-022-03498-3 (PMC9184628; doi:10.1038/s42003-022-03498-3)
Supplement: Supplementary file 3 — Description of Additional Supplementary Files [file 42003_2022_3498_MOESM3_ESM.pdf]

Description of Additional Supplementary Files

File name: Supplementary Data 1
Description: The source data underlying the plots presented in the main text.
