# Supplementary material for: AMAISE: a machine learning approach to index-free sequence enrichment
Source: Commun Biol. 2022 Jun 9;5:568. doi: 10.1038/s42003-022-03498-3 (PMC9184628; doi:10.1038/s42003-022-03498-3)
Supplement: Supplementary file 4 — Supplementary Data 1 [file 42003_2022_3498_MOESM4_ESM.docx]

**Figure 2 Data**

Figure 2, Accuracies

|  | **Host Fraction (% of all sequences)** | | | | |
| --- | --- | --- | --- | --- | --- |
| **Approach** | **1%** | **25%** | **50%** | **75%** | **99%** |
| Kraken2-H | 0.9750 | 0.9432 | 0.9087 | 0.8758 | 0.8387 |
| Centrifuge-H | 0.9105 | 0.9022 | 0.8925 | 0.8843 | 0.8718 |
| Minimap2-H | 0.9946 | 0.9595 | 0.9216 | 0.8846 | 0.8445 |
| AMAISE | 0.9889 | 0.9899 | 0.9909 | 0.9922 | 0.9940 |

Figure 2, Sensitivities

|  | **Host Fraction (% of all sequences)** | | | | |
| --- | --- | --- | --- | --- | --- |
| **Approach** | **1%** | **25%** | **50%** | **75%** | **99%** |
| Kraken2-H | 0.8242 | 0.8428 | 0.8403 | 0.8416 | 0.8372 |
| Centrifuge-H | 0.8616 | 0.8752 | 0.8734 | 0.8745 | 0.8712 |
| Minimap2-H | 0.8371 | 0.8485 | 0.8465 | 0.8472 | 0.8429 |
| AMAISE | 0.9928 | 0.9940 | 0.9939 | 0.9939 | 0.9941 |

Figure 2, Specificities

|  | **Host Fraction (% of all sequences)** | | | | |
| --- | --- | --- | --- | --- | --- |
| **Approach** | **1%** | **25%** | **50%** | **75%** | **99%** |
| Kraken2-H | 0.9771 | 0.9769 | 0.9772 | 0.9782 | 0.9859 |
| Centrifuge-H | 0.9112 | 0.9113 | 0.9117 | 0.9138 | 0.9315 |
| Minimap2-H | 0.9967 | 0.9967 | 0.9968 | 0.9968 | 0.9978 |
| AMAISE | 0.9888 | 0.9885 | 0.9879 | 0.9870 | 0.9858 |

Figure 2, Classification Time (in seconds)

|  | **Host Fraction (% of all sequences)** | | | | |
| --- | --- | --- | --- | --- | --- |
| **Approach** | **1% (sec)** | **25% (sec)** | **50% (sec)** | **75% (sec)** | **99% (sec)** |
| Kraken2-H | 12.7872 | 13.0533 | 12.9895 | 13.0380 | 13.2528 |
| Centrifuge-H | 27.5637 | 37.2383 | 43.6950 | 52.0948 | 61.2333 |
| Minimap2-H | 2.0260 | 5.5175 | 8.0752 | 14.7020 | 18.8887 |
| AMAISE | 18.4837 | 18.3267 | 18.2638 | 17.7880 | 18.1587 |

Figure 2, Peak Memory Usage (GB)

|  | **Host Fraction (% of all sequences)** | | | | |
| --- | --- | --- | --- | --- | --- |
| **Approach** | **1% (GB)** | **25% (GB)** | **50% (GB)** | **75% (GB)** | **99% (GB)** |
| Kraken2-H | 4.0873 | 4.0911 | 4.0856 | 4.0830 | 4.0796 |
| Centrifuge-H | 1.5031 | 1.6539 | 1.7205 | 1.7655 | 1.8025 |
| Minimap2-H | 12.5929 | 19.6232 | 19.8712 | 19.1546 | 19.8210 |
| AMAISE | 4.6693 | 4.6532 | 4.6942 | 4.6861 | 4.6557 |

Figure 2, Storage (GB)

|  | **Host Fraction (% of all sequences)** | | | | |
| --- | --- | --- | --- | --- | --- |
| **Approach** | **1% (GB)** | **25% (GB)** | **50% (GB)** | **75% (GB)** | **99% (GB)** |
| Kraken2-H | 4.1272 | 4.1272 | 4.1272 | 4.1272 | 4.1272 |
| Centrifuge-H | 1.4867 | 1.4867 | 1.4867 | 1.4867 | 1.4867 |
| Minimap2-H | 7.6275 | 7.6275 | 7.6275 | 7.6275 | 7.6275 |
| AMAISE | 0.0030 | 0.0030 | 0.0030 | 0.0030 | 0.0030 |

**Figure 3 Data**

Figure 3a

| **Approach** | **Accuracy** | **Sensitivity** | **Specificity** | **Classification Time (sec)** | **Peak Memory Usage (GB)** | **Storage (GB)** |
| --- | --- | --- | --- | --- | --- | --- |
| Kraken2-H | 0.9812 | 0.9916 | 0.8893 | 2.0193 | 4.0605 | 4.1272 |
| Centrifuge-H | 0.9826 | 0.9977 | 0.8498 | 6.3713 | 1.5538 | 1.4867 |
| Minimap2-H | 0.9811 | 0.9906 | 0.8970 | 1.4442 | 12.4757 | 7.6275 |
| AMAISE | 0.9780 | 0.9863 | 0.9048 | 6.3823 | 3.0551 | 0.0030 |

Figure 3b, Frequencies within Each Bin of Histogram of Sequence Lengths of Synthetic Dataset

| **Right Edge of Bin (in Sequence Length)** | **Frequency of Sequence Length in Bins** |
| --- | --- |
| 82 | 323167 |
| 2432 | 229435 |
| 4782 | 134675 |
| 7132 | 85741 |
| 9482 | 57369 |
| 11832 | 40931 |
| 14182 | 30031 |
| 16532 | 21415 |
| 18882 | 15675 |
| 21232 | 11561 |

Figure 3b, Frequencies within Each Bin of Histogram of Lengths of Real Metagenomic Dataset

| **Right Edge of Bin (in Sequence Length)** | **Frequency of Sequence Length in Bins** |
| --- | --- |
| 475 | 326931 |
| 773.5 | 192987 |
| 1072 | 114403 |
| 1370.5 | 70797 |
| 1669 | 45529 |
| 1967.5 | 30908 |
| 2266 | 22003 |
| 2564.5 | 15791 |
| 2863 | 11866 |
| 3161.5 | 8889 |

Figure 3c, Percentage of Unique K-mers in Host Data

|  | **K-mer Length** | | | | |
| --- | --- | --- | --- | --- | --- |
| **Dataset** | **11** | **13** | **15** | **17** | **21** |
| Real Metagenomic Dataset | 11.6563 | 76.8820 | 97.0195 | 99.4715 | 99.7440 |
| Synthetic Dataset | 1.2995 | 13.3579 | 78.2227 | 97.5013 | 99.9496 |

**Figure 4 Data**

Figure 4, Host Accuracies

|  | **Host Fraction (% of all sequences)** | | | | |
| --- | --- | --- | --- | --- | --- |
| **Approach** | **1%** | **25%** | **50%** | **75%** | **99%** |
| Centrifuge-HM | 0.8338 | 0.8519 | 0.8495 | 0.8511 | 0.8471 |
| AMAISE + Centrifuge-M | 0.9928 | 0.9940 | 0.9939 | 0.9939 | 0.9941 |

Figure 4, Classification Time (in seconds)

|  | **Host Fraction (% of all sequences)** | | | | |
| --- | --- | --- | --- | --- | --- |
| **Approach** | **1%** | **25%** | **50%** | **75%** | **99%** |
| Centrifuge-HM | 56.4220 | 61.2167 | 62.5667 | 66.6000 | 71.4333 |
| AMAISE + Centrifuge-M | 71.9277 | 58.8457 | 45.1615 | 30.9538 | 18.7485 |

Figure 4, Peak Memory Usage (GB)

|  | **Host Fraction (% of all sequences)** | | | | |
| --- | --- | --- | --- | --- | --- |
| **Approach** | **1%** | **25%** | **50%** | **75%** | **99%** |
| Centrifuge-HM | 8.4433 | 8.5286 | 8.5936 | 8.6482 | 8.6833 |
| AMAISE + Centrifuge-M | 6.9699 | 6.9612 | 6.9496 | 6.9354 | 6.9107 |

**Figure 5 Data**

Figure 5a, Classification Time on Server (in seconds)

|  | **Host Fraction (% of all sequences)** | | | | |
| --- | --- | --- | --- | --- | --- |
| **Approach** | **1%** | **25%** | **50%** | **75%** | **99%** |
| Kraken2-H | 12.7872 | 13.0533 | 12.9895 | 13.0380 | 13.2528 |
| Centrifuge-H | 27.5637 | 37.2383 | 43.6950 | 52.0948 | 61.2333 |
| AMAISE | 18.4837 | 18.3267 | 18.2638 | 17.7880 | 18.1587 |

Figure 5a, Classification Time on Google Cloud Platform Virtual Machine (in seconds)

|  | **Host Fraction (% of all sequences)** | | | | |
| --- | --- | --- | --- | --- | --- |
| **Approach** | **1%** | **25%** | **50%** | **75%** | **99%** |
| Kraken2-H | 13.7790 | 15.0220 | 14.7195 | 16.1757 | 13.9217 |
| Centrifuge-H | 31.8795 | 46.3335 | 55.2525 | 70.4333 | 85.7000 |
| AMAISE | 44.1643 | 45.0825 | 44.1020 | 43.7307 | 44.0935 |

Figure 5b, Classification Time on Server (in seconds)

|  | **Host Fraction (% of all sequences)** | | | | |
| --- | --- | --- | --- | --- | --- |
| **Approach** | **1%** | **25%** | **50%** | **75%** | **99%** |
| Centrifuge-HM | 56.4220 | 61.2167 | 62.5667 | 66.6000 | 71.4333 |
| AMAISE + Centrifuge-M | 71.9277 | 58.8457 | 45.1615 | 30.9538 | 18.7485 |

Figure 5b, Classification Time on Google Cloud Platform Virtual Machine (in seconds)

|  | **Host Fraction (% of all sequences)** | | | | |
| --- | --- | --- | --- | --- | --- |
| **Approach** | **1%** | **25%** | **50%** | **75%** | **99%** |
| Centrifuge-HM | 64.8333 | 72.5833 | 80.4000 | 80.8500 | 88.8833 |
| AMAISE + Centrifuge-M | 106.9187 | 91.7802 | 76.0225 | 59.6842 | 48.9890 |

**Figure 6 Data**

Figure 6

| **15-mer** | **Overall Contribution of 15-mer to Host Classification Label** | **Individual Nucleotide Contributions** |
| --- | --- | --- |
| ATAAAACGCTTTTTT | -0.8305 | -0.2007, -0.0488, -0.2713, -0.1836, -0.1397, 0.0090, -0.5000, -0.4886, -0.1369, -0.2209, -0.2276, -0.1780, -0.3192, -0.3935, -0.1820 |
| ATCAAAAAGCGTTTG | -0.8270 | -0.1392, -0.1318, -0.0755, -0.1572, -0.2493, -0.1951, -0.2336, -0.2235, -0.1021, -0.5000, -0.4947, 0.1008, -0.1672, -0.0071, -0.1794 |
| CGCGATTATGAATTT | -0.8257 | -0.3117, -0.3721, -0.5000, -0.3997, -0.1499, -0.1731, -0.0186, -0.0794, 0.0114, 0.0192, -0.0557, -0.0087, -0.0388, -0.1090, -0.1788 |
| AATAAAACGCTTTTT | -0.8215 | -0.0924, -0.1397, -0.0469, -0.2717, -0.1285, -0.0970, 0.0045, -0.4640, -0.5000, -0.1301, -0.1876, -0.1068, -0.1611, -0.2977, -0.2776 |
| TTATCAAAAAGCGTT | -0.8206 | -0.1063, -0.0926, -0.1732, -0.1548, -0.0798, -0.1830, -0.2731, -0.1892, -0.1855, -0.2383, -0.1331, -0.4973, -0.5000, 0.0765, -0.2011 |
| CCTCTCTCTCTCTCA | 0.9281 | 0.2831, 0.3172, 0.1682, 0.2320, 0.1415, 0.2798, 0.1520, 0.2614, 0.1280, 0.2393, 0.1655, 0.5000, 0.2122, 0.2411, 0.1872 |
| GGCTGGAGTGCAGGA | 0.9365 | 0.5000, 0.2884, 0.3055, 0.1621, 0.2973, 0.4662, 0.2919, 0.2790, 0.1093, 0.0834, 0.1974, 0.0390, 0.3344, 0.0953, -0.0626 |
| GTCCCAGCTGGGAGG | 0.9421 | 0.0479, 0.1626, 0.4719, 0.2861, 0.3142, 0.1548, 0.2340, 0.2027, 0.2644, 0.0921, 0.2956, 0.5000, 0.3113, 0.4082, 0.1595 |
| CCCAGCTGGGAGGCT | 0.9563 | 0.5000, 0.3482, 0.3748, 0.2108, 0.3666, 0.2380, 0.3499, 0.0831, 0.3546, 0.4799, 0.3268, 0.4359, 0.2046, 0.3176, 0.0280 |
| TCCCAGCTGGGAGGC | 0.9996 | 0.2122, 0.4899, 0.2895, 0.3018, 0.1573, 0.3352, 0.2381, 0.2897, 0.0937, 0.3175, 0.5000, 0.2848, 0.3767, 0.1535, 0.2857 |

**Figure 7 Data**

Figure 7a, Accuracies

|  | **% CG in Sequences** | | | | |
| --- | --- | --- | --- | --- | --- |
| **Approach** | **< 1 %** | **Between 1 and 2%** | **Between 2 and 4%** | **Between 4 and 10%** | **> 10%** |
| Kraken2-H | 0.9733 | 0.9391 | 0.7723 | 0.8701 | 0.9889 |
| Centrifuge-H | 0.9647 | 0.9324 | 0.7468 | 0.8459 | 0.9728 |
| Minimap2-H | 0.9866 | 0.9602 | 0.7901 | 0.8792 | 0.9921 |
| AMAISE | 0.9921 | 0.9937 | 0.9838 | 0.9873 | 0.9977 |

Figure 7a, Sensitivities

|  | **% CG in Sequences** | | | | |
| --- | --- | --- | --- | --- | --- |
| **Approach** | **< 1 %** | **Between 1 and 2%** | **Between 2 and 4%** | **Between 4 and 10%** | **> 10%** |
| Kraken2-H | 0.9832 | 0.9472 | 0.6149 | 0.2051 | 0.2392 |
| Centrifuge-H | 0.9863 | 0.9648 | 0.6979 | 0.3206 | 0.4443 |
| Minimap2-H | 0.9868 | 0.9550 | 0.6098 | 0.2403 | 0.4324 |
| AMAISE | 0.9986 | 0.9988 | 0.9856 | 0.9685 | 0.9765 |

Figure 7a, Specificities

|  | **% CG in Sequences** | | | | |
| --- | --- | --- | --- | --- | --- |
| **Approach** | **< 1%** | **Between 1 and 2%** | **Between 2 and 4%** | **Between 4 and 10%** | **> 10%** |
| Kraken2-H | 0.8351 | 0.8856 | 0.9488 | 0.9930 | 0.9991 |
| Centrifuge-H | 0.6634 | 0.7172 | 0.8016 | 0.9431 | 0.9800 |
| Minimap2-H | 0.9845 | 0.9942 | 0.9921 | 0.9973 | 0.9997 |
| AMAISE | 0.9006 | 0.9594 | 0.9818 | 0.9908 | 0.9980 |

Figure 7b, Percentage of Unique K-mers in Host Data

|  | **K-mer Length** | | | | |
| --- | --- | --- | --- | --- | --- |
| **% CG in Sequences** | **11** | **13** | **15** | **17** | **21** |
| < 1% | 56.9114 | 89.1521 | 97.8229 | 99.6597 | 99.9867 |
| Between 1 and 2% | 34.6085 | 80.4213 | 96.4838 | 99.5603 | 99.9857 |
| Between 2 and 4% | 2.3841 | 47.1823 | 90.7840 | 99.0395 | 99.9631 |
| Between 4 and 10% | 0.3481 | 28.7749 | 87.9392 | 98.8212 | 99.9268 |
| > 10% | 1.0515 | 22.6110 | 68.4588 | 92.9909 | 99.7473 |
