# Supplementary material for: AMAISE: a machine learning approach to index-free sequence enrichment
Source: Commun Biol. 2022 Jun 9;5:568. doi: 10.1038/s42003-022-03498-3 (PMC9184628; doi:10.1038/s42003-022-03498-3)
Supplement: Supplementary file 5 — Reporting Summary [file 42003_2022_3498_MOESM5_ESM.pdf]

nature portfolio

Corresponding author(s): Jenna Wiens

Last updated by author(s): Mar 8, 2022

Reporting Summary

Nature Portfolio wishes to improve the reproducibility of the work that we publish. This form provides structure for consistency and transparency in reporting. For further information on Nature Portfolio policies, see our Editorial Policies and the Editorial Policy Checklist.

Statistics

For all statistical analyses, confirm that the following items are present in the figure legend, table legend, main text, or Methods section.

n/a	Confirmed	
☐	☒	The exact sample size (*n*) for each experimental group/condition, given as a discrete number and unit of measurement
☒	☐	A statement on whether measurements were taken from distinct samples or whether the same sample was measured repeatedly
☒	☐	The statistical test(s) used AND whether they are one- or two-sided<br>*Only common tests should be described solely by name; describe more complex techniques in the Methods section.*
☒	☐	A description of all covariates tested
☒	☐	A description of any assumptions or corrections, such as tests of normality and adjustment for multiple comparisons
☒	☐	A full description of the statistical parameters including central tendency (e.g. means) or other basic estimates (e.g. regression coefficient) AND variation (e.g. standard deviation) or associated estimates of uncertainty (e.g. confidence intervals)
☒	☐	For null hypothesis testing, the test statistic (e.g. *F*, *t*, *r*) with confidence intervals, effect sizes, degrees of freedom and *P* value noted<br>*Give P values as exact values whenever suitable.*
☒	☐	For Bayesian analysis, information on the choice of priors and Markov chain Monte Carlo settings
☒	☐	For hierarchical and complex designs, identification of the appropriate level for tests and full reporting of outcomes
☒	☐	Estimates of effect sizes (e.g. Cohen's *d*, Pearson's *r*), indicating how they were calculated

Our web collection on statistics for biologists contains articles on many of the points above.

Software and code

Policy information about availability of computer code

Data collection

To train and test AMAISE and baselines, we sampled sequences from reference genomes from NCBI and Nanopore reads from NCBI's Sequence Read Archive.

To sample reference genomes, we applied the following selection criteria using R and downloaded genomes that passed. For the non-host genomes, we first examined the frequencies of different types of genomic assemblies available in the NCBI RefSeq genomes database using assembly summary files. Observing that we would receive a representation of all phylogenetic lineages, we selected reference and representative assemblies that included complete and chromosomal level representation from multiple species. For viral genomes, we also selected ICTV species exemplars. We chose three host genomes to train from: the human reference genome (GRCh38.p13), the mouse reference genome (GRCm39), and the pig representative genome (Sscrofa11.1).

We masked the downloaded reference genomes' repeat regions with dustmasker with the default parameters during database creation as was recommended by the authors of Kraken2 and Centrifuge.

The Nanopore reads used to train and test AMAISE were obtained from NCBI's Sequence Read Archive and the CEPH1463 (NA12878/GM12878, Ceph/Utah pedigree) human genome reference standard on the Oxford Nanopore MinION created by Jain et al, available from the European Nucleotide Archive under accession PRJEB23027.

Data analysis

We compared AMAISE to Minimap2 (Version 2.17), Kraken2 (Version 2.1.0), and Centrifuge (Version 1.0.4). We ran each tool such that it used 16 threads for computation. For Kraken2-H, Centrifuge-H, and Minimap2-H we created indices with the human reference genome and aligned our test sets to that data. If a read in the test set was not aligned to that index, then we would consider that read to be from a microbe. For Kraken2-HM and Centrifuge-HM, we created databases with the 2951 bacterial reference genomes, 194 archaeal reference genomes, 57 fungal reference genomes, 34 protist reference genomes, and 5455 viral genomes that we used to train AMAISE as well as the human reference genome. For Kraken2-M and Centrifuge-M, we created databases with the 2951 bacterial reference genomes, 194 archaeal reference genomes, 57 fungal reference genomes, 34 protist reference genomes, and 5455 viral genomes that we used to train AMAISE.

We used six evaluation metrics to compare AMAISE to existing in silico host depletion tools: classification accuracy, sensitivity, specificity, maximum resident set size, storage requirements, and elapsed wall clock time. We calculated classification accuracy using Python library Scikit-Learn's function "accuracy_score." We calculated sensitivity and specificity by using Scikit-Learn's function "confusion_matrix" to calculate the number of true negatives, false positives, false negatives, and true positives, and then calculated sensitivity by dividing the number of true positives by the number of true positives + the number of false negatives. We calculated specificity by dividing the number of true negatives by the number of true negatives + false positives.

We used five evaluation metrics to compare AMAISE + Centrifuge-M and AMAISE + Kraken2-M to Centrifuge-HM and Kraken2-HM: host accuracy, multi-class microbial accuracy, maximum resident set size, storage requirements, and elapsed wall clock time. The accuracy on the sequences labeled as host was computed by the percentage of sequences that were correctly labeled as `Homo sapiens' out of the number of sequences with the true label `Homo sapiens.' The accuracy on the sequences labeled as microbial was computed by the weighted average of the number of sequences that were labeled as a certain microbe whose true label was that microbe.

We model our classification time and part of our maximum resident set size evaluation on the evaluation in the Kraken2 paper. We ran each host depletion method using 16 threads on the same number of reads. We used the taskset command to restrict the number of processors each method was allowed to use. We used the "/usr/bin/time -v" command to get the elapsed wall clock time and the maximum resident set size. AMAISE also used VRAM via the GPUs, so we added the MiB of VRAM that AMAISE used to the amount of RAM that it used. We calculated the amount of VRAM that AMAISE used with the command "nvidia-smi." We evaluate storage requirements using the "ls -l" command in the folders containing the existing host depletion methods' databases and indices and the folder containing AMAISE. We did not include the storage necessary for the packages needed to run the baseline methods and AMAISE.

We created the histograms that displayed these evaluation metrics in the paper using the Python libraries Numpy, Pandas, and Matplotlib.

We applied the DeepLift algorithm from Captum, Pytorch's library for Model Interpretability, to a Nanopore read set to identify the k-mers that contributed to AMAISE's classification decision. The DeepLift algorithm determines the contribution of each element of an input sequence to the final classification by approximating the gradient of the model output with respect to each element of the input in the following way. It compares the activation of each neuron in the model given the current input to its activation given a reference input. Based on the difference in values, the DeepLift algorithm computes a contribution score for each neuron and uses those contribution scores to compute the final contribution for each element of the input. Instead of using a single reference, we used 20 different references, where each reference was a permuted version of the input sequence, and we averaged the output contributions to get the final contribution of each nucleotide in the input sequence. This was found to be the best strategy for assigning input contributions to DNA sequence data.

Once we calculated the input attributions for a sequence, we summed the attributions from each nucleotide in a k-mer (where k = 15) to calculate the amount that each k-mer in the sequence contributed to the final classification decision. We stored the total attribution of each k-mer in each input sequence. After doing so, we calculated the mean attribution. We then sorted the k-mers by the mean attribution and selected the 5 k-mers that had the highest attributions and the 5 k-mers that had the lowest attributions for further analysis. We looked at k-mers where k = 15 because AMAISE's first convolution layer learns patterns of length 15.

For manuscripts utilizing custom algorithms or software that are central to the research but not yet described in published literature, software must be made available to editors and reviewers. We strongly encourage code deposition in a community repository (e.g. GitHub). See the Nature Portfolio guidelines for submitting code & software for further information.

Data

Policy information about availability of data

All manuscripts must include a data availability statement. This statement should provide the following information, where applicable:

- Accession codes, unique identifiers, or web links for publicly available datasets
- A description of any restrictions on data availability
- For clinical datasets or third party data, please ensure that the statement adheres to our policy

The reference genomes used to train AMAISE are available on NCBI. Their accession codes are available on https://gitlab.eecs.umich.edu/meerak/amaise. The test sets that we used to train and evaluate AMAISE are downloaded from NCBI's Sequence Read Archive (their accession codes are available on https://gitlab.eecs.umich.edu/meerak/amaise) and the CEPH1463 (NA12878/GM12878, Ceph/Utah pedigree) human genome reference standard on the Oxford Nanopore MinION created by Jain et al which is available from the European Nucleotide Archive under accession PRJEB23027.

Field-specific reporting

Please select the one below that is the best fit for your research. If you are not sure, read the appropriate sections before making your selection.

☒ Life sciences ☐ Behavioural & social sciences ☐ Ecological, evolutionary & environmental sciences

For a reference copy of the document with all sections, see nature.com/documents/nr-reporting-summary-flat.pdf

Life sciences study design

All studies must disclose on these points even when the disclosure is negative.

Sample size	No sample size calculations were performed. All test sets contained over 800,000 DNA sequences. This size was chosen to evaluate AMAISE and baselines' efficiency when classifying a large number of sequences.

Data exclusions	No data exclusions
Replication	We used large test sets and a large amount of microbial variation in our test sets to ensure that the results in our paper can be replicated.
Randomization	The test sets were created first to ensure maximum species variation, randomly sampling from sequences downloaded from NCBI's Sequence Read Archive. The reference genomes that we used to train AMAISE contained all the microbes in the test sets, so the training set did not need to contain variation from Nanopore sequencing technology. Once the test sets were created, from the remaining samples that were not included in the test set, the training and validation sets were created, sampling sequences at random. Then the training and validation sets were augmented by randomly sampling sequences from reference genomes.
Blinding	Blinding was not relevant to this study because there was no null hypothesis testing.

Reporting for specific materials, systems and methods

We require information from authors about some types of materials, experimental systems and methods used in many studies. Here, indicate whether each material, system or method listed is relevant to your study. If you are not sure if a list item applies to your research, read the appropriate section before selecting a response.

Materials & experimental systems

n/a	Involved in the study	
☒	☐	Antibodies
☒	☐	Eukaryotic cell lines
☒	☐	Palaeontology and archaeology
☒	☐	Animals and other organisms
☒	☐	Human research participants
☒	☐	Clinical data
☒	☐	Dual use research of concern

Methods

n/a	Involved in the study	
☒	☐	ChIP-seq
☒	☐	Flow cytometry
☒	☐	MRI-based neuroimaging
